# Supplementary material for: Physiological, Biochemical and Yield-Component Responses of Solanum tuberosum L. Group Phureja Genotypes to a Water Deficit
Source: Plants (Basel). 2021 Mar 27;10(4):638. doi: 10.3390/plants10040638 (PMC8065493; doi:10.3390/plants10040638)
Supplement: Supplementary file 1 [file plants-10-00638-s001.pdf]

Supplementary tables

Table S1. Rotated component matrix of soluble sugar content, maximum quantum of PSII photochemistry (F_v/F_m), relative chlorophyll content (CC), tuber number (TN) tuber fresh weight(TW) and relative water content (RWC) under well-watered and water deficit conditions and Drought Tolerance Index (DTI) of 104 genotypes from the Work Collection of the Potato Breeding program at the Universidad Nacional de Colombia.

Variable	Well-watered			Water deficit			Drought stress index		
	PC-1	PC-2	PC-3	PC-1	PC-2	PC-3	PC-1	PC-2	PC-3
Sucrose	0.752	0.156	-0.099	0.757	0.109	0.21	0.767	0.311	0.139
Glucose	0.823	-0.153	-0.229	0.815	0.099	0.12	0.839	0.115	0.096
Fructose	0.753	-0.223	-0.183	0.91	0.06	0.107	0.877	-0.129	0.1665
F_v/F_m	0.016	0.173	0.785	-0.305	0.509	0.593	-0.128	0.731	-0.379
CC	0.178	0.708	0.126	-0.045	0.555	-0.042	-0.203	0.595	0.414
TN	-0.345	0.406	-0.526	-0.05	0.621	-0.466	-0.351	0.051	0.538
TW	0.061	0.792	-0.237	0.124	0.595	0.527	-0.115	0.285	0.761
RWC	0.49	0.285	0.407	-0.1972	0.416	0.679	0.061	0.724	-0.341
Explain variance (eigenvalue)	2.206	1.505	1.228	2.221	1.509	1.382	2.262	1.625	1.359
Proportion of variance (%)	27.586	18.817	15.355	27.767	18.866	17.275	28.28	20.317	16.998
Cumulate variance(%)	27.586	46.403	61.759	27.767	46.634	63.91	28.28	48.598	65.596

Abbreviations: PC-1, principal component 1; PC-2, principal component 2; PC-3, principal component 3.

Table S2. Average content of soluble sugars, maximum quantum efficiency of PSII (F_v/F_m), relative chlorophyll content, tuber number, tuber weight and relative water content (RWC) under water deficit conditions of 104 genotypes from *Solanum tuberosum* Group Phureja. The genotype group from the cluster analysis is also shown for each genotype.

Genotype	Sucrose (mg $^{g-1}$ fw)	Glucose (mg $^{g-1}$ fw)	Fructose (mg $^{g-1}$ fw)	F_v/F_m	Relative chlorophyll content (SPAD unit)	Tuber number	Tuber weight (g per plant)	Relative water content (%)	Group
CCC[a]002	1,88	3,26	7,39	0,73	41,48	5,17	23,80	42,74	1
CCC003	0,97	0,83	1,51	0,70	32,58	7,50	43,98	51,76	1
CCC004	1,30	1,00	1,51	0,77	38,22	7,67	48,70	66,77	1
CCC005	2,37	4,20	5,60	0,80	33,52	5,40	28,32	63,89	1
CCC006	3,38	3,01	4,08	0,77	38,13	7,50	35,08	72,99	1
CCC007	2,29	3,58	6,89	0,78	39,90	6,67	32,43	73,05	1
CCC008	2,71	1,82	2,07	0,80	32,38	11,40	79,10	58,02	1
CCC009	3,18	3,32	5,04	0,80	37,45	8,00	42,63	65,74	1
CCC011	2,33	2,44	3,07	0,79	39,72	7,50	33,48	50,36	1
CCC013	2,20	1,29	2,33	0,79	41,37	5,50	59,00	66,63	1
CCC014	2,68	5,90	15,87	0,68	37,27	3,33	16,67	59,35	3
CCC015	4,23	4,40	5,21	0,78	46,57	10,00	68,77	44,19	3
CCC016	2,84	3,20	4,60	0,82	43,02	8,83	37,68	63,94	1
CCC017	2,46	2,39	4,34	0,77	48,07	11,83	72,12	42,56	2
CCC019	2,81	3,11	9,53	0,77	42,70	8,67	66,90	47,46	3
CCC020	2,58	4,37	9,92	0,81	49,12	7,83	55,47	54,86	3
CCC021	6,09	5,24	7,75	0,79	45,35	4,00	24,90	72,53	3
CCC023	3,09	2,53	2,87	0,78	50,80	5,33	53,07	56,65	1
CCC024	2,49	2,13	3,49	0,79	43,70	9,75	38,55	47,55	1
CCC027	5,02	4,95	6,81	0,81	44,48	4,20	28,72	52,96	3
CCC030	3,68	4,45	6,06	0,76	36,47	4,67	49,93	54,12	3
CCC031	2,46	2,84	4,36	0,79	42,00	7,83	42,63	65,23	1

CCC032	3,09	3,42	4,10	0,80	49,42	3,83	60,02	64,03	1
CCC033	3,80	2,80	4,86	0,81	45,13	8,33	36,65	50,98	1
CCC035	3,87	4,28	5,55	0,84	55,20	9,50	127,87	68,97	2
CCC037	4,16	5,91	7,10	0,80	45,22	8,67	71,48	60,84	3
CCC038	2,80	4,06	7,68	0,73	42,42	8,50	35,32	40,83	3
CCC041	5,13	6,66	12,20	0,22	29,53	3,75	64,60	18,35	4
CCC042	4,56	1,93	4,14	0,80	50,10	6,33	53,23	64,53	1
CCC043	4,38	4,05	6,59	0,81	44,68	12,17	77,67	53,96	3
CCC044	3,01	3,57	5,66	0,79	40,72	3,33	49,90	46,29	1
CCC045	2,57	2,47	2,96	0,78	44,92	6,17	47,87	52,55	1
CCC047	3,91	3,27	5,24	0,78	37,33	5,00	80,45	41,19	3
CCC051	2,16	2,04	4,47	0,84	42,12	5,00	25,27	48,39	1
CCC052	4,01	1,70	2,62	0,79	46,80	6,50	40,75	49,74	1
CCC053	4,18	6,20	11,29	0,80	39,42	6,83	67,25	62,24	3
CCC056	2,66	3,26	4,72	0,78	38,57	4,17	28,65	57,55	1
CCC057	3,35	5,15	8,98	0,80	41,88	7,17	34,08	64,25	3
CCC059	2,25	2,67	3,05	0,83	44,50	6,00	21,85	55,82	1
CCC061	3,07	2,58	7,31	0,82	42,88	3,20	10,64	62,62	1
CCC062	4,14	17,70	22,77	0,81	46,44	11,00	94,26	54,35	5
CCC063	3,29	4,46	7,94	0,79	47,27	6,50	74,30	60,53	3
CCC065	4,07	3,87	8,91	0,80	46,28	5,50	26,90	58,08	3
CCC066	4,74	4,17	6,70	0,73	37,75	7,67	33,43	62,82	3
CCC067	3,08	2,42	4,28	0,80	40,05	9,17	36,47	66,06	1
CCC069	1,51	2,43	3,65	0,79	43,93	10,50	99,35	64,48	2
CCC070	3,85	3,24	10,38	0,76	50,74	8,00	50,32	51,58	3
CCC071	2,63	4,57	9,25	0,77	40,90	14,60	102,12	45,53	2
CCC072	6,46	4,76	7,82	0,79	38,08	5,60	41,34	53,42	3
CCC073	4,20	6,54	10,15	0,78	47,08	6,17	52,82	50,79	3
CCC074	2,67	3,16	4,17	0,78	46,93	6,33	56,93	50,92	1

CCC076	10,04	6,60	33,88	0,82	41,05	5,00	25,00	51,97	5
CCC079	4,06	6,27	7,03	0,77	47,77	11,67	57,95	60,52	3
CCC080	2,32	3,60	4,88	0,82	55,30	21,33	137,40	60,68	2
CCC081	2,19	2,21	4,17	0,79	41,40	4,83	20,40	50,10	1
CCC083	3,04	4,14	4,53	0,77	49,53	9,00	57,78	50,53	3
CCC086	1,78	3,25	4,66	0,80	48,57	10,00	79,55	59,50	1
CCC087	2,53	3,76	5,03	0,74	49,23	9,67	156,13	55,14	2
CCC088	2,42	4,18	6,06	0,79	36,85	7,33	49,68	61,60	1
CCC089	2,76	2,57	2,67	0,80	51,73	5,50	26,38	71,64	1
CCC091	2,55	4,40	7,30	0,79	44,08	13,00	51,20	65,47	3
CCC093	3,54	4,93	6,12	0,77	49,07	8,50	153,28	55,83	2
CCC096	1,10	1,72	5,15	0,81	47,45	7,50	64,28	17,90	1
CCC098	2,68	2,29	1,71	0,78	44,52	4,17	28,65	63,56	1
CCC099	2,44	2,57	3,05	0,79	45,48	7,83	44,12	56,35	1
CCC101	1,63	2,79	4,78	0,74	48,82	12,67	82,60	53,74	2
CCC102	2,17	3,27	5,70	0,78	48,55	9,50	23,63	50,66	1
CCC103	3,35	5,29	7,06	0,78	47,37	5,83	61,57	68,60	3
CCC104	2,46	4,32	11,94	0,80	42,98	7,00	53,23	50,32	3
CCC106	2,28	5,72	7,38	0,80	44,48	9,50	56,13	59,13	3
CCC108	2,89	3,33	8,08	0,79	41,40	9,75	84,05	51,86	3
CCC109	1,77	3,39	4,09	0,74	43,24	7,60	34,12	47,75	1
CCC110	2,50	3,21	5,14	0,82	44,93	6,25	16,68	64,65	1
CCC112	1,14	3,28	4,51	0,77	42,92	10,17	34,90	39,05	1
CCC113	2,02	1,74	2,55	0,77	41,25	13,00	31,48	39,92	1
CCC114	1,77	5,41	7,64	0,79	40,78	7,40	15,56	66,12	1
CCC115	2,15	4,35	6,38	0,78	36,15	11,83	35,08	62,16	1
CCC116	2,32	2,80	3,66	0,80	42,35	14,00	50,05	66,34	1
CCC117	2,98	4,42	5,15	0,78	42,02	10,00	49,48	54,33	3
CCC118	3,14	4,86	4,39	0,73	34,52	9,33	53,08	47,28	3

CCC119	1,94	2,43	3,23	0,79	45,48	7,00	54,47	60,96	1
CCC120	4,15	3,35	4,64	0,78	37,70	18,67	65,88	58,54	2
CCC121	2,52	3,87	5,01	0,77	42,05	12,83	53,92	46,71	1
CCC122	3,40	4,82	9,30	0,78	41,38	13,17	77,82	65,38	3
CCC123	2,22	4,64	5,15	0,78	41,50	12,75	45,18	46,88	1
CCC124	1,38	3,97	5,65	0,74	44,17	6,17	27,25	62,43	1
CCC125	2,63	1,93	2,94	0,78	42,10	11,33	38,60	50,76	1
CCC126	2,91	4,00	6,51	0,76	39,48	10,17	67,28	63,18	3
CCC127	2,37	2,23	2,32	0,80	40,62	8,17	71,35	58,42	1
CCC128	3,31	3,18	4,82	0,80	42,90	11,17	59,07	51,33	1
CCC129	1,31	0,72	0,82	0,76	41,15	14,50	52,42	49,37	1
CCC131	4,35	2,93	6,16	0,75	113,58	11,33	41,72	50,83	2
CCC132	2,67	1,78	4,36	0,72	45,40	6,20	59,32	57,11	1
CCC133	2,86	3,71	11,75	0,73	46,70	11,50	66,78	47,00	3
CCC135	2,76	7,39	12,03	0,82	47,42	8,50	44,95	57,24	3
CCC136	7,51	3,80	16,67	0,74	43,06	30,40	63,92	58,64	2
CCC137	0,87	0,54	3,40	0,80	46,90	20,67	63,97	54,36	2
CCC138	2,39	3,42	3,77	0,80	42,58	11,67	21,83	43,88	1
CCC140	3,04	1,95	1,18	0,82	46,50	10,00	18,90	57,86	1
CCC141	3,84	6,81	11,33	0,74	33,03	4,83	21,93	31,57	3
CCC142	2,06	2,14	1,18	0,75	45,82	10,83	51,17	67,15	1
CCC143	2,91	4,45	11,98	0,75	49,55	5,83	94,30	57,43	3
CCC144	1,97	3,40	6,99	0,78	46,53	10,83	51,37	64,81	1
CCC145	2,98	4,77	3,28	0,83	39,95	5,67	33,50	73,38	1

Table S3. Average content of soluble sugars, the maximum quantum efficiency of PSII photochemistry (F_v/F_m), relative chlorophyll content, tuber number, tuber fresh weight and relative water content (RWC) under well-watered conditions of 104 genotypes from *Solanum tuberosum* Group Phureja. The genotype group from the cluster analysis is also shown for each genotype.

Genotype	Sucrose (mg g^{-1} fw)	Glucose (mg g^{-1} fw)	Fructose (mg g^{-1} fw)	F_v/F_m	Relative Chlorophyll content (SPAD unit)	Tuber number	Tuber weight (g per plant)	Relative water content (%)	Group
CCC[a]002	1,69	1,26	2,79	0,82	42,45	6,67	66,00	85,46	3
CCC003	1,92	2,03	5,38	0,81	38,60	8,50	62,55	84,25	2
CCC004	2,82	2,55	2,97	0,82	41,87	10,83	111,53	83,11	3
CCC005	1,35	0,63	1,02	0,82	38,83	9,00	53,83	82,66	1
CCC006	1,83	0,90	1,85	0,82	41,38	4,75	89,13	80,09	1
CCC007	1,68	2,67	5,36	0,83	36,48	5,83	77,20	90,67	2
CCC008	1,17	0,42	0,98	0,83	39,38	5,75	57,20	86,43	1
CCC009	2,80	1,66	3,86	0,83	34,68	9,83	90,08	85,28	2
CCC011	1,36	2,25	1,91	0,84	33,20	10,83	58,25	82,46	2
CCC013	3,57	3,75	4,37	0,83	47,00	4,00	104,85	86,43	3
CCC014	1,42	2,47	3,80	0,83	41,35	9,00	64,33	85,10	3
CCC015	1,67	0,94	2,03	0,83	45,13	4,00	113,25	85,35	3
CCC016	1,37	1,47	2,45	0,83	40,32	7,33	102,55	85,39	3
CCC017	2,03	2,11	3,47	0,81	39,45	13,83	130,27	88,48	3
CCC019	1,33	0,82	1,24	0,80	37,27	12,50	154,02	83,96	1
CCC020	2,14	2,21	3,92	0,83	43,37	8,67	93,37	85,49	3
CCC021	2,12	2,66	3,27	0,81	43,70	7,50	37,08	77,79	2
CCC023	3,06	1,45	1,46	0,82	44,98	8,83	99,22	82,46	3
CCC024	1,50	1,48	2,58	0,82	42,80	6,00	42,65	78,03	2
CCC027	2,27	2,97	3,37	0,83	38,44	4,60	47,82	77,37	2
CCC030	1,37	1,40	5,72	0,83	37,50	5,00	61,15	79,04	2
CCC031	1,95	1,46	2,34	0,81	36,53	5,75	89,43	83,54	2

CCC032	2,55	2,34	3,12	0,82	48,25	3,75	102,73	77,50	3
CCC033	1,33	0,94	1,40	0,81	42,03	5,50	69,83	78,02	1
CCC035	2,86	0,67	0,75	0,83	51,38	4,50	162,58	83,44	3
CCC037	3,56	1,56	4,00	0,83	42,20	5,50	117,40	82,74	3
CCC038	1,12	0,99	1,40	0,84	39,43	12,33	81,03	84,65	1
CCC041	0,53	0,81	3,76	0,83	35,05	3,25	122,33	75,87	1
CCC042	0,70	0,62	0,75	0,83	37,68	5,50	80,25	81,53	1
CCC043	2,54	2,56	2,95	0,81	39,43	7,75	130,85	82,67	3
CCC044	1,27	0,83	1,14	0,83	36,55	1,75	103,78	83,97	1
CCC045	1,74	0,84	1,12	0,82	43,13	7,50	102,88	82,44	1
CCC047	0,89	0,86	3,50	0,82	40,23	7,00	139,53	77,46	1
CCC051	0,83	0,87	1,25	0,82	37,05	4,50	66,87	79,39	1
CCC052	0,65	0,75	1,81	0,81	37,80	3,50	79,83	71,77	1
CCC053	1,65	1,34	2,01	0,81	33,90	3,25	69,70	78,37	2
CCC056	0,94	0,59	0,75	0,82	38,63	3,00	24,83	72,93	1
CCC057	1,99	0,82	1,29	0,84	40,28	5,75	55,40	84,05	1
CCC059	2,01	1,19	0,64	0,82	43,00	4,50	20,78	78,06	1
CCC061	1,82	0,59	3,36	0,83	44,48	8,00	39,12	74,11	1
CCC062	1,33	0,36	0,59	0,82	43,35	4,50	29,85	83,17	1
CCC063	0,83	0,38	0,79	0,84	42,50	4,25	128,55	83,90	1
CCC065	0,52	0,38	0,78	0,82	40,02	6,00	51,24	81,17	1
CCC066	1,30	0,73	1,38	0,82	42,50	4,25	41,28	81,29	1
CCC067	1,78	1,17	3,85	0,80	37,20	8,75	62,23	82,07	2
CCC069	5,00	1,61	1,26	0,84	42,35	10,00	75,20	80,94	3
CCC070	1,11	0,84	2,61	0,84	41,45	5,00	78,65	83,04	1
CCC071	1,63	0,89	3,23	0,81	38,72	13,00	119,78	82,47	1
CCC072	2,55	1,53	2,59	0,80	37,10	8,00	44,40	79,09	2
CCC073	1,52	3,57	1,23	0,83	43,70	6,00	116,25	84,61	3
CCC074	1,23	0,68	0,99	0,83	46,08	5,25	108,00	82,14	1

CCC076	2,24	2,79	3,56	0,83	34,93	3,00	14,90	81,30	2
CCC079	2,23	2,14	2,88	0,82	41,78	6,50	79,75	78,30	2
CCC080	1,59	1,90	1,85	0,83	47,53	10,75	136,85	82,51	3
CCC081	1,44	2,04	2,29	0,82	37,70	6,00	61,75	85,22	2
CCC083	1,95	0,89	1,22	0,82	41,68	10,00	89,28	83,56	1
CCC086	1,69	0,86	1,22	0,83	43,03	8,75	107,73	78,87	1
CCC087	1,26	0,74	2,11	0,81	45,00	8,75	195,40	84,41	3
CCC088	1,20	2,75	3,01	0,82	36,08	5,25	92,80	81,08	2
CCC089	1,82	1,97	1,54	0,83	48,18	8,75	37,00	79,03	3
CCC091	0,94	1,00	0,85	0,84	39,40	10,00	54,54	82,72	1
CCC093	1,53	2,67	3,86	0,82	46,05	10,67	247,68	79,93	3
CCC096	1,04	1,14	2,39	0,82	40,35	7,25	63,38	76,15	1
CCC098	0,47	0,40	0,46	0,83	38,15	6,25	29,75	77,94	1
CCC099	1,50	0,82	2,17	0,82	41,80	6,00	102,00	73,89	1
CCC101	0,58	1,63	1,62	0,82	45,72	15,50	106,38	74,53	1
CCC102	1,99	1,13	1,20	0,82	44,47	11,83	85,02	76,40	1
CCC103	0,87	0,93	2,94	0,84	41,60	3,67	68,60	81,88	1
CCC104	1,91	1,20	1,34	0,83	39,13	6,00	93,45	75,81	1
CCC106	1,57	1,65	1,78	0,82	37,77	12,00	91,98	76,70	1
CCC108	1,76	1,41	1,70	0,82	35,45	5,00	77,60	82,74	2
CCC109	2,31	1,12	1,25	0,82	40,65	9,25	59,98	79,82	1
CCC110	1,16	0,68	2,54	0,82	42,03	11,75	54,55	74,23	1
CCC112	0,90	0,93	1,13	0,81	37,17	9,67	74,47	78,72	1
CCC113	0,60	1,13	1,77	0,84	35,85	10,67	76,50	75,79	1
CCC114	1,13	2,64	3,61	0,81	35,63	6,00	26,20	74,15	2
CCC115	0,95	0,93	1,14	0,84	31,78	11,00	49,55	75,82	1
CCC116	0,63	0,95	1,65	0,83	40,35	15,83	72,90	71,61	1
CCC117	0,68	0,73	2,16	0,82	36,53	9,33	85,32	87,09	1
CCC118	1,09	0,59	1,87	0,82	35,28	10,33	91,58	85,05	1

CCC119	1,60	1,65	1,61	0,83	39,85	7,83	76,78	79,39	1
CCC120	0,70	0,43	2,12	0,82	38,63	16,83	156,13	75,57	1
CCC121	0,94	0,81	1,03	0,83	38,92	12,83	110,38	73,43	1
CCC122	0,76	0,51	1,33	0,83	37,23	6,00	88,13	72,80	1
CCC123	0,64	0,20	0,27	0,82	40,98	12,75	106,00	82,68	1
CCC124	1,12	1,18	2,39	0,81	41,08	6,33	55,28	74,26	1
CCC125	1,27	1,11	1,23	0,82	38,75	11,17	77,98	73,08	1
CCC126	1,08	0,81	0,89	0,82	37,82	9,83	77,82	78,06	1
CCC127	1,19	0,69	0,74	0,82	40,38	4,25	127,45	78,25	1
CCC128	0,55	0,45	1,93	0,83	38,72	8,83	108,43	78,12	1
CCC129	2,66	2,12	2,53	0,81	34,88	7,50	61,88	75,44	2
CCC131	0,80	0,71	1,11	0,81	35,55	11,17	64,70	77,45	1
CCC132	1,62	1,33	1,79	0,84	38,83	8,75	88,03	80,81	1
CCC133	0,69	1,12	1,16	0,82	43,08	12,67	104,03	77,66	1
CCC135	1,54	0,72	1,84	0,82	45,27	7,00	77,68	79,32	1
CCC136	2,59	2,01	2,32	0,79	42,78	11,50	81,58	76,64	2
CCC137	1,83	0,68	1,22	0,83	42,88	19,50	160,93	76,88	1
CCC138	1,22	0,88	1,31	0,82	38,25	6,25	48,25	73,66	1
CCC140	0,39	0,25	0,43	0,83	37,97	15,33	51,17	80,63	1
CCC141	2,71	3,33	4,74	0,83	33,02	4,00	33,07	81,80	2
CCC142	1,27	0,72	0,68	0,82	37,50	16,25	121,53	73,63	1
CCC143	1,83	1,37	2,05	0,83	42,65	5,83	139,03	87,62	3
CCC144	1,87	2,16	2,91	0,83	40,63	5,50	108,58	77,11	2
CCC145	0,97	2,48	1,43	0,81	41,83	5,83	69,63	74,14	2

Table S4. Average drought Tolerance Index (DTI) for soluble sugars content, maximum quantum efficiency of PSII photochemistry (F_v/F_m), relative chlorophyll content, tuber number, tuber weight and relative water content (RWC) of 104 genotypes from *Solanum tuberosum* Group Phureja. The genotype group from the cluster analysis is also shown for each genotype.

Genotype	Sucrose (mg g^{-1} fw)	Glucose (mg g^{-1} fw)	Fructose (mg g^{-1} fw)	F_v/F_m	Relative chlorophyll content (SPAD unit)	Tuber number	Tuber weight (g per plant)	Relative water content (%)	Group
CCC[a]002	1,327	2,318	4,753	0,886	1,091	0,540	0,217	0,570	2
CCC003	0,771	0,949	1,878	0,836	0,779	1,000	0,380	0,680	2
CCC004	1,528	1,440	1,034	0,937	0,991	1,303	0,750	0,866	2
CCC005	1,338	1,488	1,318	0,977	0,806	0,762	0,210	0,824	2
CCC006	2,585	1,524	1,738	0,935	0,977	0,559	0,432	0,912	2
CCC007	1,605	5,394	8,519	0,962	0,902	0,610	0,346	1,034	4
CCC008	1,315	0,437	0,467	0,975	0,790	1,028	0,625	0,783	2
CCC009	3,718	3,118	4,486	0,977	0,805	1,234	0,530	0,875	2
CCC011	1,325	3,109	1,354	0,977	0,817	1,275	0,269	0,648	2
CCC013	3,278	2,731	2,346	0,967	1,204	0,345	0,854	0,899	2
CCC014	1,583	8,233	13,902	0,835	0,955	0,471	0,148	0,788	4
CCC015	2,937	2,344	2,438	0,962	1,302	0,627	1,075	0,588	2
CCC016	1,621	2,661	2,598	1,001	1,074	1,016	0,533	0,852	2
CCC017	2,084	2,844	3,464	0,926	1,175	2,568	1,297	0,588	2
CCC019	1,556	1,445	2,714	0,908	0,986	1,699	1,422	0,622	2
CCC020	2,300	5,466	8,964	0,998	1,319	1,065	0,715	0,732	4
CCC021	5,390	7,886	5,842	0,953	1,228	0,471	0,127	0,880	4
CCC023	3,940	2,073	0,962	0,936	1,416	0,739	0,727	0,729	2
CCC024	1,561	1,783	2,075	0,951	1,159	0,918	0,227	0,579	2
CCC027	4,737	8,318	5,291	0,992	1,059	0,303	0,190	0,639	4
CCC030	2,107	3,519	7,990	0,938	0,847	0,366	0,421	0,667	2

CCC031	1,993	2,348	2,350	0,953	0,950	0,707	0,526	0,850	2
CCC032	3,292	4,525	2,946	0,970	1,477	0,226	0,851	0,774	2
CCC033	2,112	1,488	1,569	0,967	1,175	0,719	0,353	0,621	2
CCC035	4,616	1,615	0,959	1,021	1,757	0,671	2,869	0,898	1
CCC037	6,178	5,202	6,555	0,977	1,182	0,748	1,158	0,785	4
CCC038	1,305	2,264	2,478	0,907	1,036	1,645	0,395	0,539	2
CCC041	1,127	3,035	10,570	0,265	0,641	0,191	1,091	0,217	3
CCC042	1,339	0,673	0,720	0,983	1,169	0,546	0,590	0,821	2
CCC043	4,627	5,860	4,477	0,969	1,091	1,479	1,403	0,696	4
CCC044	1,596	1,674	1,490	0,966	0,922	0,092	0,715	0,607	2
CCC045	1,861	1,180	0,761	0,936	1,200	0,726	0,680	0,676	2
CCC047	1,449	1,597	4,231	0,938	0,930	0,549	1,549	0,498	2
CCC051	0,753	1,008	1,287	1,017	0,967	0,353	0,233	0,599	2
CCC052	1,094	0,715	1,094	0,950	1,096	0,357	0,449	0,557	2
CCC053	2,876	4,678	5,238	0,963	0,828	0,348	0,647	0,761	4
CCC056	1,044	1,081	0,819	0,949	0,923	0,196	0,098	0,655	2
CCC057	2,788	2,373	2,670	0,993	1,045	0,646	0,261	0,843	2
CCC059	1,884	1,791	0,451	1,005	1,185	0,424	0,063	0,680	2
CCC061	2,334	0,862	5,664	1,006	1,182	0,402	0,057	0,724	2
CCC062	2,284	3,650	3,107	0,982	1,247	0,777	0,388	0,705	2
CCC063	1,136	0,959	1,449	0,984	1,244	0,433	1,318	0,792	2
CCC065	0,876	0,837	1,593	0,973	1,147	0,518	0,190	0,736	2
CCC066	2,564	1,732	2,132	0,886	0,994	0,511	0,190	0,797	2
CCC067	2,286	1,601	3,793	0,952	0,923	1,258	0,313	0,846	2
CCC069	3,147	2,216	1,064	0,983	1,152	1,647	1,031	0,814	2
CCC070	1,771	1,536	6,254	0,938	1,303	0,627	0,546	0,668	2
CCC071	1,783	2,311	6,894	0,921	0,981	2,977	1,688	0,586	2
CCC072	6,865	4,123	4,672	0,930	0,875	0,703	0,253	0,659	4
CCC073	2,667	13,179	2,883	0,955	1,275	0,580	0,847	0,671	4

CCC074	1,368	1,225	0,952	0,963	1,340	0,522	0,849	0,653	2
CCC076	9,354	10,431	27,812	0,995	0,888	0,235	0,051	0,659	4
CCC079	3,771	7,570	4,663	0,938	1,236	1,190	0,638	0,740	4
CCC080	1,537	3,865	2,084	1,003	1,628	3,598	2,595	0,781	1
CCC081	1,318	2,551	2,208	0,964	0,967	0,455	0,174	0,666	2
CCC083	2,473	2,095	1,278	0,928	1,279	1,412	0,712	0,659	2
CCC086	1,255	1,586	1,308	0,983	1,294	1,373	1,183	0,732	2
CCC087	1,326	1,562	2,445	0,892	1,372	1,327	4,211	0,726	1
CCC088	1,208	6,496	4,208	0,954	0,823	0,604	0,636	0,779	2
CCC089	2,095	2,866	0,948	0,983	1,544	0,755	0,135	0,883	2
CCC091	1,001	2,496	1,428	0,977	1,076	2,039	0,385	0,845	2
CCC093	2,262	7,424	5,444	0,933	1,400	1,422	5,240	0,696	1
CCC096	0,475	1,107	2,840	0,987	1,186	0,853	0,562	0,213	2
CCC098	0,527	0,519	0,180	0,961	1,052	0,409	0,118	0,773	2
CCC099	1,532	1,192	1,525	0,955	1,178	0,737	0,621	0,650	2
CCC101	0,393	2,569	1,786	0,896	1,382	3,080	1,213	0,625	1
CCC102	1,801	2,097	1,581	0,946	1,337	1,763	0,277	0,604	2
CCC103	1,216	2,780	4,787	0,969	1,221	0,336	0,583	0,876	2
CCC104	1,952	2,924	3,692	0,974	1,042	0,659	0,687	0,595	2
CCC106	1,489	5,352	3,021	0,966	1,041	1,788	0,713	0,708	2
CCC108	2,118	2,648	3,175	0,957	0,909	0,765	0,900	0,670	2
CCC109	1,706	2,138	1,176	0,904	1,089	1,103	0,282	0,595	2
CCC110	1,210	1,240	3,012	0,989	1,169	1,152	0,126	0,749	2
CCC112	0,427	1,726	1,171	0,916	0,988	1,542	0,359	0,480	2
CCC113	0,504	1,111	1,040	0,961	0,916	2,175	0,332	0,472	2
CCC114	0,834	8,066	6,355	0,944	0,900	0,697	0,056	0,765	4
CCC115	0,853	2,281	1,679	0,969	0,712	2,042	0,240	0,736	2
CCC116	0,608	1,498	1,390	0,988	1,059	3,477	0,504	0,741	2
CCC117	0,848	1,814	2,568	0,944	0,951	1,464	0,583	0,738	2

CCC118	1,430	1,617	1,896	0,888	0,754	1,513	0,671	0,628	2
CCC119	1,296	2,270	1,200	0,961	1,123	0,860	0,577	0,755	2
CCC120	1,209	0,823	2,270	0,945	0,902	4,929	1,420	0,690	1
CCC121	0,981	1,774	1,188	0,935	1,014	2,584	0,821	0,535	2
CCC122	1,075	1,390	2,844	0,954	0,954	1,239	0,947	0,743	2
CCC123	0,591	0,528	0,315	0,947	1,053	2,550	0,661	0,605	2
CCC124	0,641	2,652	3,108	0,894	1,124	0,613	0,208	0,723	2
CCC125	1,392	1,214	0,830	0,944	1,011	1,985	0,415	0,579	2
CCC126	1,306	1,825	1,340	0,922	0,925	1,568	0,723	0,770	2
CCC127	1,176	0,876	0,395	0,971	1,016	0,544	1,255	0,713	2
CCC128	0,762	0,807	2,148	0,977	1,029	1,547	0,884	0,626	2
CCC129	1,448	0,870	0,479	0,901	0,889	1,706	0,448	0,581	2
CCC131	1,454	1,171	1,579	0,897	2,501	1,985	0,373	0,614	1
CCC132	1,807	1,342	1,802	0,896	1,092	0,851	0,721	0,720	2
CCC133	0,822	2,342	3,138	0,882	1,246	2,285	0,959	0,570	2
CCC135	1,766	2,999	5,098	0,989	1,330	0,933	0,482	0,708	2
CCC136	8,109	4,313	8,909	0,873	1,141	5,484	0,720	0,701	4
CCC137	0,661	0,207	0,958	0,977	1,246	6,322	1,421	0,652	1
CCC138	1,219	1,690	1,141	0,964	1,009	1,144	0,145	0,504	2
CCC140	0,489	0,272	0,116	0,999	1,094	2,405	0,133	0,728	2
CCC141	4,336	12,801	12,372	0,908	0,676	0,303	0,100	0,403	4
CCC142	1,093	0,876	0,185	0,906	1,064	2,762	0,858	0,771	2
CCC143	2,229	3,434	5,652	0,913	1,309	0,534	1,810	0,785	2
CCC144	1,537	4,143	4,689	0,957	1,171	0,935	0,770	0,780	2
CCC145	1,208	6,694	1,081	0,984	1,035	0,519	0,322	0,849	2

Supplementary Figures

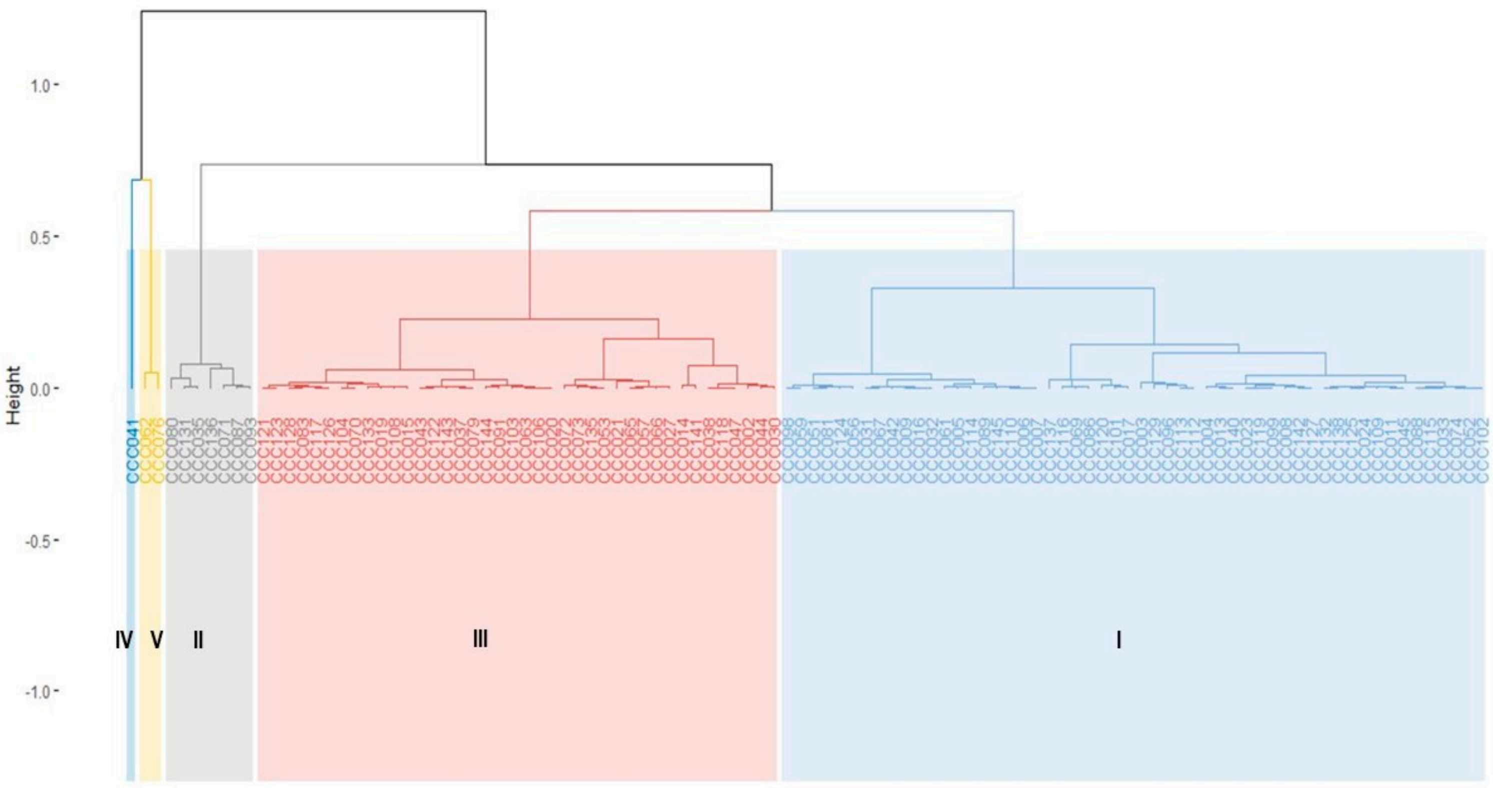

Figure S1. Factor-based hierarchical cluster analysis dendrogram using Ward's method showing 104 genotypes from the Work Collection of the Potato Breeding program at the Universidad Nacional de Colombia of *Solanum tuberosum* Group Phureja under water deficit conditions. The group numbers correspond to those given in Figure 1a.

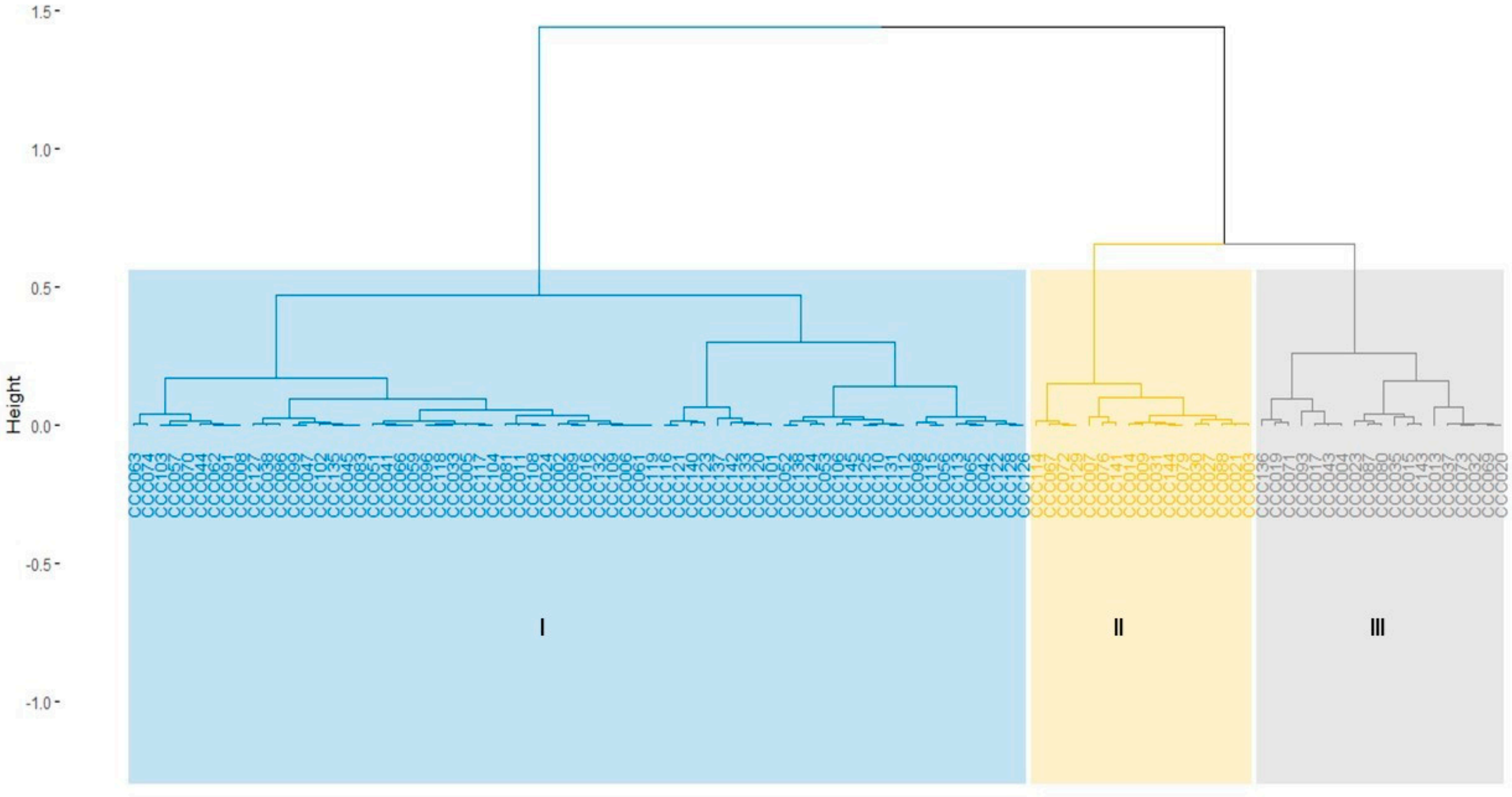

Figure S2. Factor-based hierarchical cluster analysis dendrogram using Ward's method showing 104 genotypes from the Work Collection of the Potato Breeding program at the Universidad Nacional de Colombia of *Solanum tuberosum* Group Phureja under well-water conditions. The group numbers correspond to those given in Figure 1b.

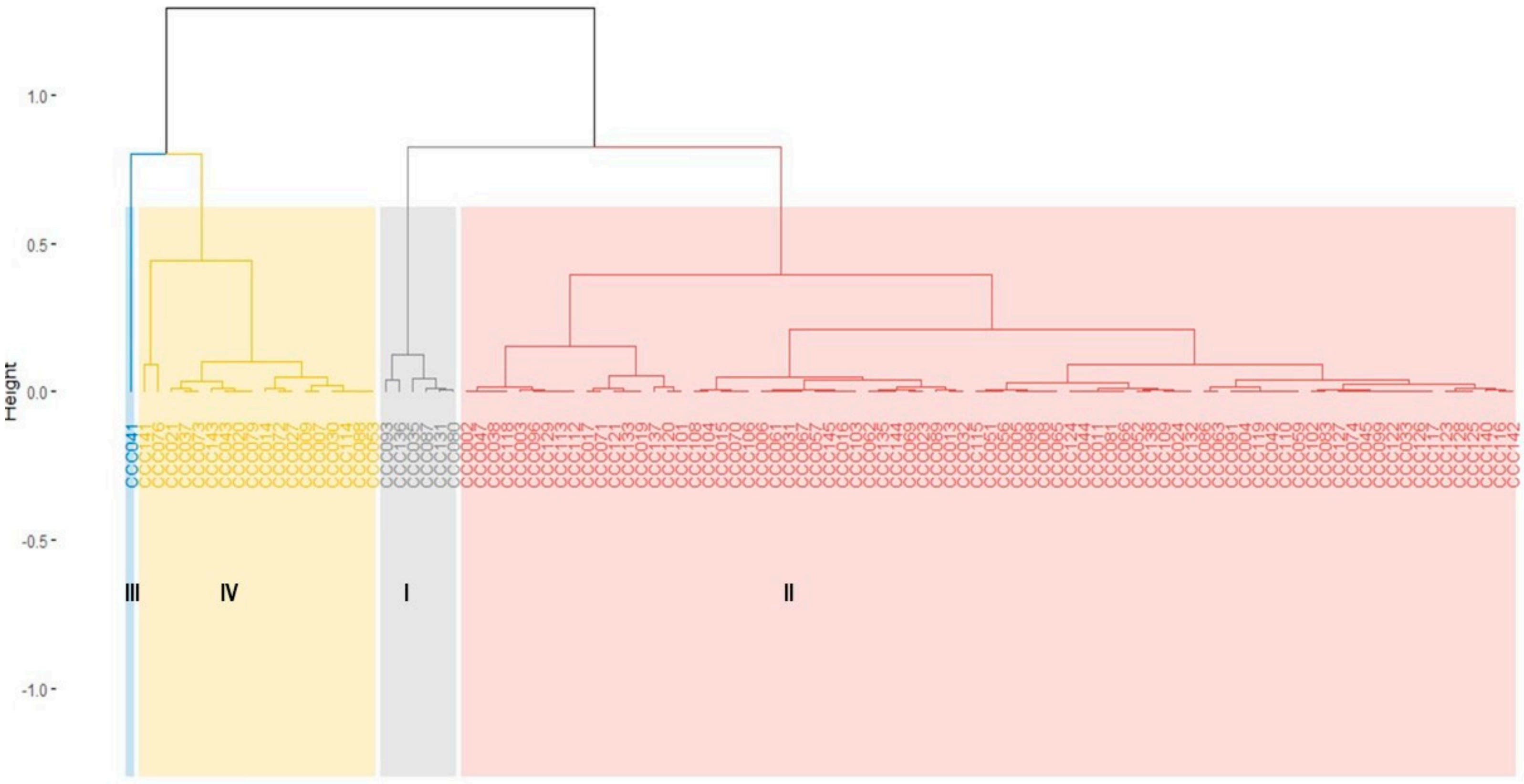

Figure S3. Factor-based hierarchical cluster analysis dendrogram using Ward's method showing 104 genotypes from the Work Collection of the Potato Breeding program at the Universidad Nacional de Colombia of *Solanum tuberosum* Group Phureja of Drought Tolerance Index (DTI). The group numbers correspond to those given in Figure 1c.
